# Blast traumatic brain injury and serum inflammatory cytokines: a repeated measures case-control study among U.S. military service members

**DOI:** 10.1186/s12974-019-1624-z

**Published:** 2020-01-13

**Authors:** Jennifer Rusiecki, Lynn I. Levin, Li Wang, Celia Byrne, Jayasree Krishnamurthy, Ligong Chen, Zygmunt Galdzicki, Louis M. French

**Affiliations:** 1 Department of Preventive Medicine and Biostatistics, F. Edward Hébert School of Medicine, Uniformed Services University, Bethesda, MD USA; 2 Statistics and Epidemiology Branch, Walter Reed Army Institute of Research, Silver Spring, MD USA; 3 Department of Pediatrics, F. Edward Hébert School of Medicine, Uniformed Services University, Bethesda, MD USA; 4 Department of Anatomy, Physiology, and Genetics, F. Edward Hébert School of Medicine, Uniformed Services University, Bethesda, MD USA; 5 National Intrepid Center of Excellence, Walter Reed National Military Medical Center, Bethesda, MD USA

**Keywords:** Traumatic brain injury, TBI, Cytokines, Inflammation, Operation Iraqi freedom, Operation enduring freedom, Protein, Microarray, Military

## Abstract

**Background:**

There is a paucity of human data on exposure to blast traumatic brain injury (bTBI) and the corresponding systemic cytokine immune response at later time points (i.e., months, years) post-injury.

**Methods:**

We conducted a repeated measures, case-control study, examining associations of serum levels of pro- and anti-inflammatory cytokines, measured both pre- and post-deployment with having mild and moderate/severe bTBI. Utilizing serum from the Department of Defense Serum Repository cytokines were measured via an ELISA-based array for 15 cytokines. We compared pre- vs. post-levels among mild cases, moderate/severe cases, and controls and carried out case-control comparisons, using paired *t-* tests and generalized linear models.

**Results:**

The average time between bTBI and post-deployment/bTBI serum among cases was 315.8 days. From pre- to post-deployment/bTBI, levels of interleukin 8 (IL-8) were decreased among both mild cases (*μ* = − 83.43 pg/ml; s.e. = 21.66) and moderate/severe cases (*μ* = − 107.67 pg/ml; s.e. = 28.74 pg/ml), while levels increased among controls (*μ* = 32.86 pg/ml; s.e. = 30.29). The same pattern occurred for matrix metallopeptidase 3 (MMP3), with levels decreasing for moderate/severe cases (*μ* = − 3369.24 pg/ml; s.e. = 1701.68) and increasing for controls (*μ* = 1859.60 pg/ml; s.e. = 1737.51) from pre- to post-deployment/bTBI. Evidence was also suggestive of case-control differences, from pre- to post-deployment/bTBI for *interleukin 1* alpha (IL-1α), interleukin 4 (IL-4), and interleukin 6 (IL-6) among moderate/severe cases.

**Conclusion:**

The findings of this longitudinal study indicate that in the chronic phase of bTBI, levels of IL-8 and MMP3 may be substantially lower than pre-injury. These results need confirmation in other studies, potentially those that account for treatment differences, which was not possible in our study.

## Background

During more than 10 years of the global war on terror (2000–2011), approximately 290,000 US military personnel suffered traumatic brain injury (TBI); nearly 70% of these were due to explosive blast exposure [1, 2]. Blast-associated TBI (bTBI) comprises four injury methods: primary (blast overpressure waves), secondary (trauma from shrapnel/objects), tertiary (head trauma with blunt impact from blast wind), and quaternary (thermal burns, toxic chemicals) [3–5]. Primary non-penetrating blast injuries result from the direct effects of complex pressure waves generated by an explosion, known as blast overpressure (BOP) injuries [6]. Primary blast is exceedingly rare, and bTBI usually has secondary and/or tertiary components to it [7, 8]. In contrast, non-penetrating, non-blast TBI are closed head injuries such as sports-related concussions or blunt force trauma [9]. Although explosive, non-penetrating bTBI shares many clinical features with non-blast TBI [10] and the symptoms often appear the same clinically; there are other features, including neuroimaging and histopathology, as well as animal study results that suggest important differences [10–14]. Taken together, these findings support the view that bTBI may be a separate (unique) form of TBI, with distinct characteristics and etiology.

Inflammation plays a central role in the pathogenesis of secondary injury after TBI [15, 16]. Injury activates cells in the central nervous system such as glial cells, microglia, and astrocytes and causes secretion of inflammatory cytokines, chemotactic cytokines, and glycoproteins (including the metallopeptidase (MMP) family of proteins). This drives the increased deposit of parenchymal and peripheral immune cells in the target brain region with the compromised blood brain barrier (BBB) [17, 18] Cells in the periphery also produce cytokines and chemokines that are involved in both local and systemic immune responses [16, 18–21]. The release of pro- and anti-inflammatory cytokines varies, based on the timing after injury [22, 23], with molecular changes activating further downstream systemic immune responses, resulting in both reactive and restorative inflammation processes [16, 21, 24].

Clinical research on serum cytokine levels and TBI severity (mild, moderate, and severe) has mainly centered on the acute phase following a TBI, defined as 1–7 days post-trauma [23, 25, 26]. Circulating levels of a number of inflammatory cytokines such as interleukin-1β (IL-1 β), interleukin-6 (IL-6), interleukin-8 (IL-8), interleukin-10 (IL-10), and tumor necrosis factor-α (TNF-α) have been examined [16, 18, 27–30]. Only a handful of studies measured the systemic inflammatory response in human populations during the sub-acute phase, roughly 7–30 days or 14 days to 3 months [23, 31, 32] or later during the chronic period/recovery phase, loosely defined as 3 months to 1 year post-TBI [33].

An understanding of the pathophysiology and cytokine response to bTBI comes primarily from experimental blast injury models [34, 35]. Several different experimental animal models of blast-induced TBI, including studies of single blast exposures [36], repeated blast exposures [37], and blast-associated polytrauma [38], have examined inflammatory serum markers. The translation of results, however, from animal models of blast injury to the clinical setting has been problematic. Several challenges have been described including lack of consistency of results across experimental studies, reproducibility, and difficulty in developing experimental models that reflect the conditions of bTBI found in humans. These differences most likely reflect difficulties in determination of local BBB failures and/or increase in BBB permeability due to mechanical disruption or inflammatory molecules targeting endothelial cells.

Murine studies have shown that inflammatory markers might reflect a variable degree of glymphatic flux and BBB disruption following a TBI [39, 40]. Moreover, glymphatic and meningeal lymphatic systems have been shown to be present in humans [41–43].

There is a paucity of human data on the systemic cytokine immune response during the acute phase [26, 44] or during later time points specific for bTBI [45]. Better characterization of the cytokine response in peripheral serum during recovery post-bTBI would help clarify the systemic inflammatory processes during the chronic phase, may provide better monitoring of the recovery or rehabilitative period, and may possibly identify and evaluate therapeutic interventions [46–48]. Clearly, there is a need to understand the changes in circulating cytokines before and after the occurrence of bTBI and how these changes differ from levels among individuals who have not been diagnosed with a bTBI. For these reasons, we conducted a case-control study to examine serum levels of pro- and anti-inflammatory cytokines measured both prior to and during the chronic (recovery) phase after a mild, moderate, or severe bTBI diagnosis, compared with serum levels from pre- and post-deployment/bTBI among military personnel without a diagnosis of bTBI.

## Materials and method

### Study population

This longitudinal, case-control study included bTBI cases and controls with a pre-deployment and post-deployment serum sample archived in the Department of Defense (DoD) Serum Repository. This repository houses more than 62 million serum samples from more than 10 million US military service members, dating back to the late 1980s [49]. For the current study, non-penetrating bTBI cases were identified via the Defense Veterans Brain Injury Center (DVBIC) clinical database, a comprehensive database of all TBI patients treated at Walter Reed Army Medical Center between February 2003 and August 2010. Identified cases were categorized as mild, moderate, or severe, based on DoD/Veterans Administration (VA) criteria [50]. TBI presence and severity was determined through a clinical assessment involving an interview by a credentialed provider, radiologic findings, review of medical records (including point of injury records when available), and gathering of collateral information (including descriptions by third party observers when available). For cases selected for our study, personal identifiers were encrypted and sent, along with limited affiliated clinical data, from DVBIC to the Armed Forces Health Surveillance Branch (AFHSB) for linkage with DoD Serum Repository samples. Clinical data abstracted for each case included: date of injury, data on severity and nature of extra-cranial injuries (e.g., neck and cervical, face, thorax and thoracic spine, abdomen and lumbar, and extremities), and source of blast injury.

Inclusion criteria for cases were based on age (less than 40 years at the start of first OEF/OIF deployment) and date of injury (after the start of the service member’s first OEF/OIF deployment, but prior to the start of any subsequent deployments). Cases were excluded if they ever had at least one inpatient encounter or two outpatient encounters for any cancer as per the International Classification of Diseases, 9th Edition (ICD-9) codes for neoplasms (codes 140–239), with the exception of non-melanoma skin cancer. Additionally, exclusions for cases were having had at least one inpatient or two outpatient encounters for schizophrenia (ICD-9 code 295) or bi-polar disorders (ICD-9 codes 296.0, 296.4–.8) any time after the end date of their first OEF/OIF deployment. These exclusions were made because of the potential for cytokine levels to be influenced by carcinogenic processes and psychological conditions. Only cases with a pre-deployment serum specimen collected less than 1 year before the start of their first OEF/OIF deployment and a post-deployment (synonymous with post-bTBI) serum sample were included. The pre- and post-samples meeting these criteria and collected closest to the start and end dates, respectively, of the first OEF/OIF deployment were selected. Since mild cases comprised the majority of our bTBI study population, selection of mild cases was based on preferentially, selecting subjects with the shortest duration between end date of the first OEF/OIF deployment and the date of the post-serum specimen draw date. All moderate bTBI cases that met the inclusion/exclusion criteria were included in the study. Because of the small number of severe bTBI cases, all severe cases identified by the DVBIC database were included in the study population regardless of whether they met the inclusion/exclusion criteria of other pre-existing conditions with the exception that the designated pre- and post-deployment serum specimens were available.

The number of cases identified included 93 mild, 38 moderate, and 19 severe bTBI, for a total of 150 cases. For 1 of the 93 mild cases and 6 of the 19 severe cases, date of post-deployment/bTBI blood draw was found to be erroneous (i.e., prior to date of injury), so these samples were excluded from our analyses. However, the pre-deployment samples for these cases were retained. Given the small number of severe bTBI cases, we combined the moderate and severe cases into one group for analysis (e.g., moderate/severe).

A common set of controls was used for the analysis of mild cases and moderate/severe cases. Eligible controls were required to have had at least one OEF/OIF deployment and be less than 40 years of age at the start of their first OEF/OIF deployment. We excluded from the potential pool of controls any service members listed in the original DVBIC TBI clinical case database or any service member with a medical encounter for intracranial injuries (ICD-9 codes 850–854.19). Additionally, we excluded potential controls who ever had at least one inpatient or two outpatient encounters for the ICD-9 category, neoplasms (ICD-9 codes 140–239), with the exception of non-melanoma skin cancer or at least one inpatient or two outpatient encounters for schizophrenia (ICD-9 code 295) or bi-polar disorders (ICD-9 codes 296.0, 296.4–.8) any time after the end date of their first OEF/OIF deployment. Criteria for selection of pre-and post-samples used for cases was also applied for controls, resulting in selection of 50 randomly selected controls for the study, based on frequency matching to the case population by age, sex, and race (white, black, other).

Diagnosis of post-traumatic stress disorder (PTSD) was also assessed for each case and control. Two outpatient encounters of ICD-9 code 309.81 in the first diagnostic position, with the first outpatient diagnosis occurring within 4 to 12 months after the end date of the first OEF/OIF deployment and the second diagnosis occurring within 2 years after the end date of the first OEF/OIF deployment, defined PTSD.

### Cytokine measurement

Cytokine analysis was performed on the serum samples using Quantibody Human Cytokine Array 1 platform from Raybiotech, Inc. (Atlanta, GA), which utilizes multiplexed sandwich ELISA-based technology to determine the concentration of multiple cytokines simultaneously.

The Quantibody array was custom made for our study using the following 15 cytokines: nerve growth factor β (β-NGF), interleukin (IL) 1α, IL1β, IL4, IL6, IL8, IL10, IL13, IL17, monocyte chemoattractant protein-1 (MCP-1), matrix metallopeptidase 3 (MMP-3), matrix metallopeptidase 9 (MMP-9), transforming growth factor β-1 (TGFβ-1), TNFα, and TNFβ. At the time of experiment, slides were equilibrated to room temperature for 20–30 min and air dried for another 1–2 h.

A standard glass slide array was spotted with 16 wells of identical cytokine antibody. In this study, 30 μl of serum, together with positive and negative controls, were arrayed in quadruplicate. Standard cytokines and samples were assayed on each array simultaneously. The lyophilized cytokine standards were reconstituted and serial dilutions prepared using the sample diluent. The concentration of this cytokine standard was predetermined and provided along with the array kit to generate a standard curve in order to determine the concentration of each cytokine in the experimental sample. Sample diluent alone served as a negative control. The blocking of the slide was done by adding 100 μl of the sample diluent into each well of the glass slide and incubated for 30 min at room temperature to block the slides. The blocking buffer was decanted. The standard cytokines of eight different dilutions of samples of 100 μl volume were added into each well and the arrays incubated at 4 °C overnight with constant shaking. The following day, samples were decanted and wells were washed five times with 150 μl of 1× wash buffer I and two times with wash buffer II for 5 min each wash with gentle shaking at room temperature. Then, 80 μl of diluted detection antibody was added to each well and incubated at room temperature for 1–2 h with gentle shaking followed by washing with wash buffers I and II as before. Following this, 80 μl of Cy3 equivalent dye-conjugated streptavidin was added to each well and the slides were covered with foil and incubated for 1 h at room temperature with shaking. Wells were then washed with 150 μl of wash buffer I for five times at room temperature with gentle shaking. After the last wash, the slide was disassembled, removed from the gasket, and placed in the slide holder containing wash buffer1. The slides were incubated for 15 min and then washed with wash buffer 2, air-dried, and stored in dark at 4 °C. The slides were imaged with a laser scanner equipped with a Cy3 wavelength such as Axon GenePix, and data extraction was performed by Raybiotech, using microarray Quantibody Q-Analyzer data computation software for quantitative data analysis. The concentration of each cytokine in a sample was measured by comparing signals of unknown samples to the standard curve to determine the concentration of cytokine in each sample, and the final results were provided as pg/ml for further analysis.

A small number of samples were not measureable (i.e., three post-deployment/bTBI samples from controls) and some samples had readings above the maximum detectable level (i.e., one mild case pre-deployment, two mild cases post-deployment/bTBI, and one moderate case pre-deployment). These samples were, therefore, excluded from further analyses. Additionally, the array did not provide results for TNFβ (all zero values), and the majority of readings for MMP-9 were below the detection limit, precluding us from evaluating these markers. Samples were randomly ordered on plates by case/control status, and all laboratory personnel were blinded to case status. Intra-assay coefficients of variation (CV) were calculated for each slide, based on duplicate positive controls, and the maximum CV detected was 7%.

### Statistical analyses

As mentioned above, given the small number of severe bTBI cases, we conducted analyses for moderate and severe cases combined. Using paired *t* tests, we first compared pre- and post-deployment/bTBI mean cytokine levels among cases and among controls. Using generalized linear models (GLM), we determined mild bTBI case-control differences in mean cytokine levels, pre-deployment and post-deployment/bTBI, with adjustment for age, the only demographic factor that significantly differed between mild cases and controls. As no demographic factors differed between moderate/severe cases and controls, we used a simple *t* test for comparisons of mean cytokine levels between these cases and controls. Finally, we estimated the mean change in pre-post deployment cytokine levels for cases versus the change in pre-post cytokine levels for controls, using analysis of variance (ANOVA), adjusting for age in the comparisons including mild cases. We also conducted analyses stratified by age (< 25, ≥ 25 years), length of deployment (≤ 215, > 215 days (median length of deployment)), extracranial injury (with, without), PTSD (with, without), and time between injury and serum sample collection for various time cut-points (< 3 months, ≥ 3 months; < 6 months, ≥ 6 months; < 252 days, ≥ 252 days (median); < 365 days, ≥ 365 days). Because of the study sample size, we were unable to stratify by more lag time periods between injury and serum sample collection than two at a given time. We carried out sensitivity analyses to evaluate if our findings persisted after excluding cases with a PTSD diagnosis and after excluding cases without an extra-cranial injury.

Given the skewed distribution of the serum cytokine data, we evaluated analyses using log-transformed cytokine measures. Since the final results using the original, non-log transformed cytokine measurements were similar to the log-transformed data, we present the non-transformed data results in the paper.

SAS procedures *t* test, GLM, and ANOVA were used for the data analysis (SAS Version 9.3) [51]. This study was approved by the Institutional Review Boards at the Uniformed Services University of the Health Sciences and the Walter Reed Army Medical Center.

## Results

### Characteristics of cases and controls

The final sample sizes, after consideration of the noted exclusions, included 91 mild cases, 37 moderate cases, 19 severe cases, for a total of 147 cases and 50 controls for the analysis of pre-deployment samples, and 90 mild cases, 38 moderate cases, 13 severe cases, for a total of 141 cases and 47 controls for analyses of the post-deployment/bTBI samples. Baseline characteristics of the study population are presented in Table 1. All cases combined and controls did not differ by age, gender, and race, since controls were frequency matched to cases on these demographic factors. When we evaluated baseline characteristics for the two case groups (mild; moderate/severe) separately, however, mild bTBI cases were statistically significantly younger than controls, but there were no statistically significant age differences between moderate/severe cases and controls. No controls had a diagnosis of PTSD, while about 25% of cases had a PTSD diagnosis based on ICD-9 coded health encounter data. Most mild cases (85%) had some type of extra-cranial injury in addition to a traumatic brain injury. No significant differences existed between the two case groups and controls with respect to time between pre-deployment serum collection and deployment start date or length of deployment; the average time between injury onset and post-serum draw was 315.8 (sd = 261.1) days among all cases post-deployment/bTBI, 281.8 (sd = 240.3) days for mild cases, and 377.0 (sd = 287.4) days among moderate/severe cases. Among controls, the mean time between end of deployment and serum draw post-deployment was 192.4 (sd = 280.2) days.

**Table 1:**
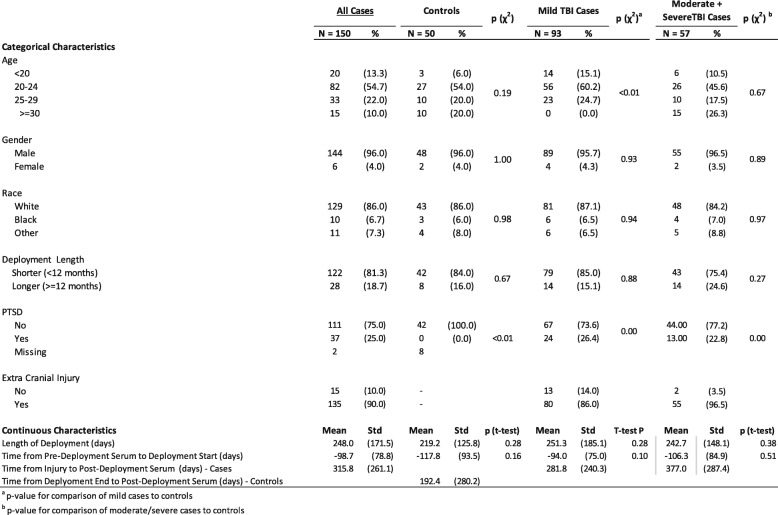
Baseline characteristics of study population of cases and controls

^a^*p* value for comparison of mild cases to controls

^b^*p* value for comparison of moderate/severe cases to controls

### Pre- and post-deployment comparisons

Table 2 compares pre-deployment and post-deployment/bTBI mean cytokine levels separately for controls, mild cases, and combined moderate/severe cases, using paired *t* tests. The IL-8 cytokine levels declined from pre- to post-serum samples for both mild cases (pre, 155.34 pg/ml; post, 71.92 pg/ml; *p* < 0.01) and for moderate/severe cases (pre, 186.01 pg/ml; post, 78.34 pg/ml; p < 0.01). Among moderate/severe cases, TGFβ1 cytokine levels also declined from pre- to post- for moderate/severe cases (pre, 4739.94 pg/ml; post, 2873.00 pg/ml; *p* = 0.05). Similar declining patterns, albeit non-statistically significant, were found among moderate/severe cases for IL-6 (pre, 52.10 pg/ml; post, 20.50 pg/ml; *p* = 0.08) and for MMP3 (pre, 21,679.6 pg/ml; post, 18,310.36 pg/ml; *p* = 0.07), pre- to post-. There were no statistically significant differences from pre- to post-deployment for controls, although IL-8 levels tended to be higher in controls’ post-deployment serum samples.

**Table 2:** Comparisons of mean cytokine levels pre- and post-deployment/bTBI for controls, mild cases, and moderate/severe cases, via paired t-test

Gene/locus	Comparison	Control				Mild				Moderate+Severe			
		N	mean	*s.e.*	*p*-value	N	mean	*s.e.*	*p*-value	N	mean	*s.e.*	*p*-value
βNGF	Pre	47	89.72	22.39		89	70.45	18.64		50	63.54	10.69	
	Post		50.57	9.35	0.10		66.00	15.98	0.84		56.14	14.28	0.70
IL-1ɑ	Pre	47	15.61	3.34		89	29.65	5.95		50	29.41	6.54	
	Post		23.77	4.88	0.11		21.33	4.39	0.20		19.49	3.47	0.18
IL-1β	Pre	47	11.71	4.77		89	15.87	5.97		50	13.94	4.86	
	Post		13.27	4.74	0.81		6.05	1.61	0.12		5.96	1.97	0.15
IL-4	Pre	47	4.91	0.88		89	5.22	1.04		50	5.67	1.34	
	Post		7.42	1.60	0.15		4.72	0.98	0.65		4.07	0.96	0.25
IL-6	Pre	47	39.69	14.86		89	34.76	9.19		50	52.10	16.69	
	Post		44.82	9.75	0.77		24.94	3.95	0.32		20.50	5.44	0.08
IL-8	Pre	45	95.04	22.78		88	155.34	18.99		50	186.01	29.72	
	Post		127.90	26.69	0.28		71.92	11.66	<0.01		78.34	17.08	<0.01
IL-10	Pre	47	10.62	1.77		89	10.17	1.86		50	9.64	1.28	
	Post		9.38	1.85	0.58		10.08	1.81	0.95		10.00	1.94	0.87
IL-13	Pre	47	65.04	12.11		89	64.09	13.72		50	74.76	13.06	
	Post		54.35	11.91	0.43		56.56	13.08	0.44		49.74	8.79	0.12
IL-17	Pre	47	63.89	10.84		89	66.67	10.85		50	78.87	11.47	
	Post		78.76	19.05	0.45		81.13	16.26	0.27		63.12	11.78	0.29
MCP1	Pre	47	105.45	8.73		89	120.95	7.34		50	111.17	6.94	
	Post		116.17	8.30	0.40		128.71	9.98	0.54		107.22	8.99	0.70
MMP3	Pre	47	20,732.20	1,297.38		85	21,995.44	966.14		49	21,679.60	1,144.74	
	Post		22,591.80	1,428.18	0.33		20,043.55	971.06	0.10		18,310.36	1,235.37	0.07
TGFβ1	Pre	47	5,097.67	999.86		89	5,378.17	943.02		50	4,739.94	774.48	
	Post		4,649.21	1,279.54	0.79		4,663.20	1,163.21	0.50		2,873.00	647.87	0.05
TNFɑ	Pre	47	682.54	96.21		89	644.05	85.58		50	702.38	107.64	
	Post		690.78	127.97	0.96		706.96	89.89	0.59		528.07	84.81	0.20

### Case-control comparisons of cytokine change, pre- to post-deployment

Table 3 shows the pre- and post-deployment/bTBI mean differences in cytokine levels for cases versus controls. Consistent with findings presented in Table 2, mean serum levels of IL-8 decreased pre- to post-deployment/bTBI for mild cases (− 83.43) and moderate/severe cases (− 107.67), but increased slightly among controls (+ 32,86), *p* < 0.01 for both comparisons. The only other statistically significant difference was found for MMP3 levels among moderate/severe cases (− 3369.24) versus controls (+ 1859.60), *p* = 0.03; a similar, non-statistically significant pattern was found among mild cases. Marginally significant case-control differences were also found for IL-1α and IL-4 in both mild cases and moderate/severe cases and for IL-6 among moderate/severe cases. Taken together, these results showed a similar pattern of decreasing cytokine levels among cases and marginally increasing levels among controls, comparing pre- to post-deployment/bTBI samples. These findings are further elucidated in Additional file 1: Table S1, which presents case-control comparisons both pre- and post-deployment/bTBI. Here, we show that post-deployment/bTBI controls’ cytokine levels were greater than cases’ for IL-6 (*p*_mild_ = 0.02; *p*_moderate/severe_ = 0.01), IL-8 (*p*_mild_ = 0.01; *p*_moderate/severe_ = 0.04), and MMP3 (*p*_moderate/severe_ = 0.03). An additional significant difference found was that post-deployment/bTBI controls’ cytokine levels were greater than cases’ for IL-1β (*p*_mild_ = 0.05; *p*_moderate/severe_ = 0.09). It should be noted that pre-deployment cases had higher levels than controls for IL-8 (*p*_mild_ = 0.05; *p*_moderate/severe_ = 0.01). All other comparisons pre-deployment showed no difference between cases and controls.

**Table 3:** Results of ANOVA with Single Model comparing means of pre-post cytokine difference for cases versus controls

Gene	Comparison	Mild bTBI Cases^a^				Moderate+Severe bTBI Cases			
		N	mean	*(s.e.)*	*p*-value	N	mean	*(s.e.)*	*p*-value
βNGF	Case	89	-4.44	19.24		50	-7.39	25.67	
	Control	47	-39.15	26.47	0.29	47	-39.15	26.47	0.39
IL-1ɑ	Case	89	-8.33	5.58		50	-9.92	7.45	
	Control	47	8.16	7.68	0.08	47	8.16	7.68	0.09
IL-1β	Case	89	-9.81	5.33		50	-7.98	7.11	
	Control	47	1.56	7.33	0.21	47	1.56	7.33	0.35
IL-4	Case	89	-0.50	1.11		50	-1.60	1.49	
	Control	47	2.51	1.53	0.11	47	2.51	1.53	0.06
IL-6	Case	89	-9.82	11.62		50	-31.60	15.51	
	Control	47	5.13	15.99	0.45	47	5.13	15.99	0.10
IL-8	Case	88	-83.43	21.66		50	-107.67	28.74	
	Control	45	32.86	30.29	<0.01	45	32.86	30.29	<0.01
IL-10	Case	89	-0.10	1.52		50	0.36	2.03	
	Control	47	-1.24	2.09	0.66	47	-1.24	2.09	0.58
IL-13	Case	89	-7.54	10.38		50	-25.03	13.85	
	Control	47	-10.69	14.28	0.86	47	-10.69	14.28	0.47
IL-17	Case	89	14.46	12.88		50	-15.76	17.19	
	Control	47	14.87	17.73	0.99	47	14.87	17.73	0.22
MCP1	Case	89	7.76	10.69		50	-3.95	14.26	
	Control	47	10.72	14.71	0.87	47	10.72	14.71	0.47
MMP3	Case	85	-1,951.88	1,292.01		49	-3,369.24	1,701.68	
	Control	47	1,859.60	1,737.51	0.08	47	1,859.60	1,737.51	0.03
TGFβ1	Case	89	-714.97	1,006.08		50	-1,866.94	1,342.28	
	Control	47	-448.46	1,384.46	0.88	47	-448.46	1,384.46	0.46
TNFɑ	Case	89	62.92	109.85		50	-174.31	146.55	
	Control	47	8.25	151.16	0.77	47	8.25	151.16	0.39

^a^Adjusted for age

### IL-8 further analysis

We further investigated findings for IL-8 by evaluating IL-8 pre- and post-deployment/bTBI mean differences for cases versus controls, stratified by age, length of deployment, evidence of extra-cranial injury, PTSD diagnosis, and time between injury and blood sample draw (Additional file 1: Table S2). Analyses stratified by age groups showed that the decreases for both mild and moderate/severe cases post-injury and increase for controls post-deployment were more pronounced for subjects younger than 25 years of age (mild cases − 85.87 pg/ml, controls + 41.46 pg/ml, *p* = 0.01; moderate/severe cases − 161.38 pg/ml, controls + 41.46 pg/ml, *p* = 0.01). For both mild cases and moderate/severe cases, mean differences were more pronounced for those with shorter (≤ 215 days) deployments (mild cases − 108.35, controls 78.92, *p* < 0.01; moderate/severe cases − 116.08, controls 78.92, *p* < 0.01). Additionally, the decreases for both mild and moderate/severe cases post-injury appeared to be more pronounced for cases with PTSD. There were no apparent differences in mean levels for those with an extra-cranial injury and without. For the lag time between bTBI diagnosis and post-deployment/bTBI serum collection, we saw a pattern of mild cases with a shorter time interval (i.e., < 3 months) having a greater reduction in cytokine levels than those with a longer time interval (≥ 3 months). We also noted similar patterns for other stratifications of time between injury and serum collection (i.e., for < 6 months vs. ≥ 6 months, etc.). For the longer lag periods between injury and serum collection (i.e., 252, 365 days), the difference between cases’ pre−/post-changes and controls’ pre−/post-changes was reduced, and by 365 days, the case/control difference was no longer statistically significant. For moderate/severe cases, there were still statistically significant differences between cases and controls beyond 365 days.

**Fig. 1:**
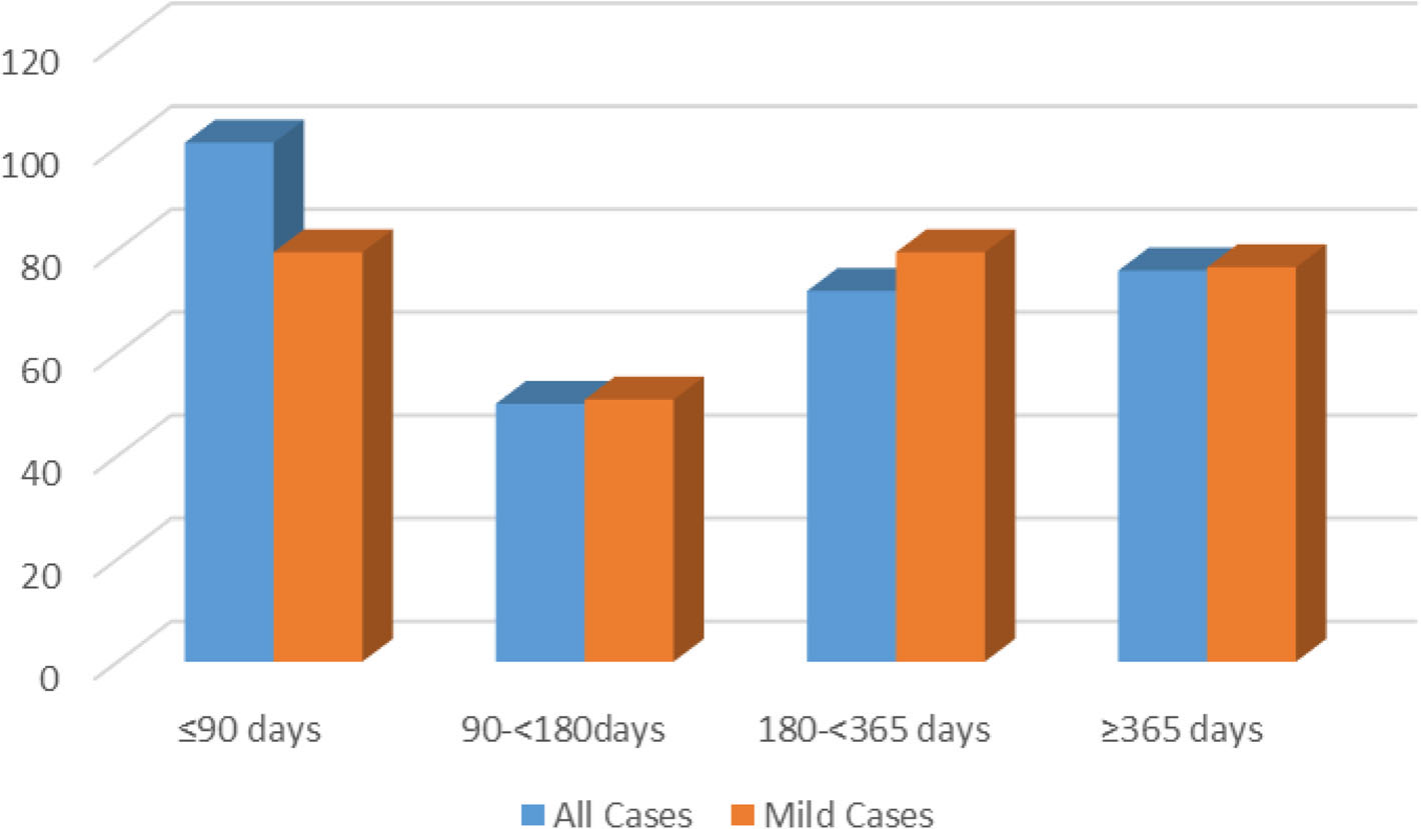
Mean post-deployment/bTBI serum IL-8 levels for all cases and for mild cases within groups of time between bTBI and serum sample collection

Figure 1 presents mean levels of IL-8 among all cases and among mild cases at various lag times between bTBI diagnosis and post-deployment/bTBI serum collection: ≤ 90 days, 90 to < 180 days, 180 to < 365 days, and ≥ 365 days. Each bar represents different groups of cases, thus not an illustration of serial measures, rather a presentation of the levels of cases within each of these lag time categories. In general, post-deployment/bTBI IL-8 levels appear to be quite stable over time for all cases and specifically for mild cases. We do not present results for moderate/severe cases, since numbers were small after stratifying into these four groups.

## Discussion

In this longitudinal study, we found that circulating serum levels of IL-8 significantly decreased from pre- to post-deployment/bTBI for both mild and moderate/severe bTBI cases. We also found that post-deployment/bTBI, both mild and moderate/severe cases, had lower IL-8 levels than controls and that the change in levels pre- to post- were significantly different between cases and controls, being more pronounced for moderate/severe cases, a difference driven more strongly by the decline in cases’ levels than the increase in controls’ levels. Intriguingly, we found that pre-deployment, controls’ IL-8 levels were significantly lower than both mild and moderate/severe cases’ levels, though controls’ levels did not significantly change from pre- to post-deployment. Decreased IL-8 serum levels were more pronounced among younger mild and mod/severe bTBI cases, those with shorter deployments, and those with PTSD. It is unclear why these sub-groups had more pronounced decreases in IL-8, and this should potentially be explored in future studies with larger sample sizes in these groups. With increasing lag time between injury and serum measurement, the pre-/post-difference between cases and controls was reduced for mild cases but remained significantly different for moderate/severe cases. We also observed a statistically significant reduction in MMP3 levels among moderate/severe bTBI cases compared with controls in pre- versus post-deployment/bTBI samples. Although differences were not statistically significant at *α* ≤ 0.05, similar patterns were found for IL-1α, IL-4, and IL-6. To our knowledge, this is the first human study focused on bTBI which includes both pre- and long-term post-injury cytokine measures.

The peripheral inflammatory response that is activated in response to injury is mediated by the complex release of cytokines which facilitate and inhibit inflammatory responses [18, 21, 52]. IL-β is a pro-inflammatory cytokine whose involvement in neuroinflammation during the acute phase post-injury has been well-described. IL-8 is a chemokine also known as CXCL8 that recruits inflammatory cells to the site of injury. IL-4 is considered an anti-inflammatory cytokine that can downregulate the production of other proinflammatory cytokines. IL-6 has been extensively studied in TBI, albeit in the acute phase, and is considered to be a singular marker of inflammation [53]. MMPs, and in particular, MMP3 and MMP9, are upregulated during TBI, because of BBB disruption, and are involved in neuroinflammation and cell death [54]. It is unclear, however, whether inflammation persists over time as measured by serum cytokine levels in the setting of TBI or bTBI [52].

While some studies have examined peripheral cytokine levels soon after bTBI [26, 44], very limited data are available to compare with our study results that describe the systemic inflammatory response during the chronic/recovery phase post-bTBI or even post-TBI. Kumar and colleagues [33] examined several inflammatory cytokines with serum specimens obtained at 2-week intervals for the first 6 months and at 12 months post-injury among severe TBI cases and controls. Several combinations of cytokines were analyzed and in conjunction with poor outcomes at 6 months and 12 months, as measured by the Glasgow Outcome Scale (GOS) scores. Overall, elevated levels of IL-1β, IL-6, IL-8, and TNF-α were seen over a 3-month period following severe TBI; however, that study did not include pre-injury serum cytokines levels, thus precluding comparison to pre-injury levels. Interestingly, the study also showed that levels of IL-8 increased among cases from 6 months to 12 months. Devoto and colleagues reported elevated serum levels of IL-6, IL-10, and TNF-α among bTBI cases compared with controls during the recovery period, post-injury [45]. Serum samples were obtained within 16 months after deployment, but the timing of the specimen collection during recovery phase in relation to the bTBI diagnosis was not described. A recent study evaluated the changes in cytokine concentrations pre- and post- a blast-related training program, finding that those exposed to higher blast pressures had higher cytokine (IL-6) concentrations but that those concentrations rebounded to baseline levels the day after the training blast exposure [26].

The idea that elevated serum levels of inflammatory cytokines might persist during the chronic phase following a bTBI originates from studies showing that elevated cytokines levels measured during the acute phase post-TBI were predictive of poor outcomes up to 1 year post-injury, as measured by the GOS scores [33, 55]. The results presented here are not in agreement with these studies. However, the average time between injury and post-injury serum draw among cases in our study was approximately 10.5 months (315.8 days), and relatively few samples from cases in our study were among those with less than 1 month between injury and post-injury serum draw (8 mild cases; 5 moderate/severe cases).

The attenuation in bTBI cases of pro-inflammatory cytokines as IL-6, IL-8 and IL1-α found in our study may suggest an ongoing neuroprotective mechanism related to the suppression of NF-κB activation and/or inhibition of p38 mitogen-activated protein kinase (MAPK) [56, 57]. Regarding the attenuation of MMP3, this cytokine belongs to a large family of endoproteinases primarily involved in turnover and remodeling of the extracellular matrix, and its expression in the brain is largely restricted to astrocytes [17], possibly impacting astrocyte interactions with blood vessels with astroglial scaring, considered as the hallmark of blast injury [14]. Changes in peripheral blood composition due to a post-blast chronic progressive or healing/neuroprotective condition may also affect the detected cytokine levels, as previously reported [58–60]. The cytokine stability in serum may be affected by storage conditions [61, 62], but according to one study [63], freeze thaw cycles should not significantly affect levels of some of the cytokines measured in our study (TNF-α and IL-6). The results reported here suggest that long-term cytokine profiles may not reflect changes seen immediately following blast results; however, we cannot completely exclude comorbidity with other clinical conditions and we cannot rule out the influence of drug therapy influencing these levels.

This study has several strengths and limitations which should be pointed out. We had unique access to serum samples, both prior to bTBI and prior to OEF/OIF deployment and post-deployment/bTBI, which allowed comparisons of baseline levels of circulating cytokine levels with serum levels determined after an initial bTBI diagnosis. Other strengths of this study are the relatively large sample size of 150 cases of mild and moderate/severe bTBI identified from clinical records and the ability to measure cytokine levels during the recovery period as serum samples were identified over a 12-month period following a bTBI diagnosis. Paired analyses revealed that controls’ levels did not differ significantly between pre- and post-deployment. It is important to include a control group in this type of study, and this control group likely represents the cohort which gave rise to these cases, given both cases and controls are from the active duty military with OEF/OIF deployment. The serum samples were handled using standard laboratory procedures, assayed on the same ELISA plate, with laboratory personnel blinded to case-control status. Of note, the CV% for each cytokine was low, in the range of 7% or less. Limitations include the possibility that some unaccountable differences exist between the bTBI cases and controls, in addition to brain injury, that affected the cytokine levels to produce these observed results. Of note, we also did not have access to information on treatment modalities for the mild or moderate/severe bTBI cases, the effects of which could have altered the detected cytokine levels in the post-deployment/bTBI serum samples. However, some of the most commonly used treatment interventions in TBI, such as psychostimulants, antidepressants, and anticonvulsants, operate via binding to dopamine transporters, increasing dopamine levels in the brain, affecting levels of norepinephrine and serotonin or enhancing inhibitory control mediated by neurotransmitters [64]. It is unclear if inflammatory cytokine levels are influenced by these common treatments; however, this should be considered in future studies. Headache, a common symptom in TBI patients is sometimes treated with non-steroidal anti-inflammatory or triptans (5-HT agonists). The extent to which that was a factor in the current study is unclear. Similarly, as described above, sleep disturbance, common in TBI patients, may affect the cerebral spinal fluid (CSF) and lymphatic/glymphatic clearance and, as shown in murine models, may reduce cytokine levels in serum following a TBI [65, 66]. However, our study did not include data on sleep disturbance. Increased inflammatory cytokines and MMPs have been implicated in migraine, and we also did not have data in our study about migraine. These factors could significantly impact the outcome of our analysis. In addition, a PTSD diagnosis was determined from medical encounter databases rather than from clinical records, resulting in the possibility of some misclassification of the PTSD diagnosis. Finally, given our small sample size, stratification by lag time between injury and serum sample was limited.

## Conclusions

In conclusion, we found an indication that circulating cytokine levels in mild and moderate/severe bTBI cases differed from controls during the recovery phase after bTBI in pre-to post-deployment/bTBI serum samples, with IL-8 levels decreased in mild and moderate/severe bTBI and MMP3 levels decreased in moderate/severe bTBI. There was limited evidence of reduced serum levels of IL-1ɑ, IL-4, and IL-6 during the recovery period. Our study results represent the chronic stage post-bTBI and should be interpreted as such, since we did not have access to samples immediately after the bTBI and had few samples within 1 month after injury. In summary, we may speculate that in the months following a bTBI, brain clearance is slowed down to the extent that serum biomarker levels are primarily regulated by a reduced glympahtic flow kinetic, previously reported in Alzheimer disease in animal models and following sleep deprivation, whereas in hours or days after a TBI, direct impact to the nervous tissue might underlie the primary source of serum biomarkers. Given that human studies of brain tissue are highly invasive, identifying a low-invasive biomarker for TBI would be of great clinical value. Although it is unclear whether inflammatory cytokine levels are influenced by conventional TBI treatments used in the military, the results of our study should be interpreted with caution, since we did not have data on treatment modalities for the cases in our study population. Additional research in other study populations focusing on bTBI and the chronic phase and including post-diagnostic treatment data will be required to further elucidate the association between serum cytokine levels with longer term follow-up after a TBI diagnosis. If confirmed, these results may suggest that in the long run TBI may slow down either the production of cytokines or the glymphatic clearance of those cytokines, thereby reducing their levels in serum. Detailed characterization of the cytokine milieu in peripheral serum during recovery post-bTBI would elucidate the systemic inflammatory process, may help to monitor the recovery or rehabilitative period, and may possibly identify and evaluate therapeutic interventions.

## Supplementary information

**Additional file 1: Table S1.** Mean differences between cases controls, pre deployment and post deployment. **Table S2.** Stratified analyses of ANOVA with single model comparing IL-8 means of pre-post cytokine difference for cases versus controls

## Data Availability

The datasets generated during and/or analyzed during the current study are not publicly available due to their being based on US military data but are available from the corresponding author on reasonable request.

## Abbreviations

AFHSB: Armed Forces Health Surveillance Branch
ANOVA: Analysis of variance
BBB: Blood brain barrier
BOP: Blast overpressure
bTBI: Blast traumatic brain injury
CV: Coefficient of variation
DoD: Department of Defense
DVBIC: Defense Veterans Brain Injury Center
GOS: Glasgow Outcome Score
ICD-9: International Classification of Diseases, 9th Edition
IL: Interleukin
MCP: Monocyte chemoattractant protein
MMP: Matrix metallopeptidase
NGF: Nerve growth factor
PTSD: Post-traumatic stress disorder
SAS: Statistical Analysis Software, Inc.
sd: Standard deviation
se: Standard error
TBI: Traumatic brain injury
TGF: Transferring growth factor
VA: Veterans Administration
