# Supplementary figures and images for: Blast traumatic brain injury and serum inflammatory cytokines: a repeated measures case-control study among U.S. military service members
Source: J Neuroinflammation. 2020 Jan 13;17:20. doi: 10.1186/s12974-019-1624-z (PMC6958571; doi:10.1186/s12974-019-1624-z)

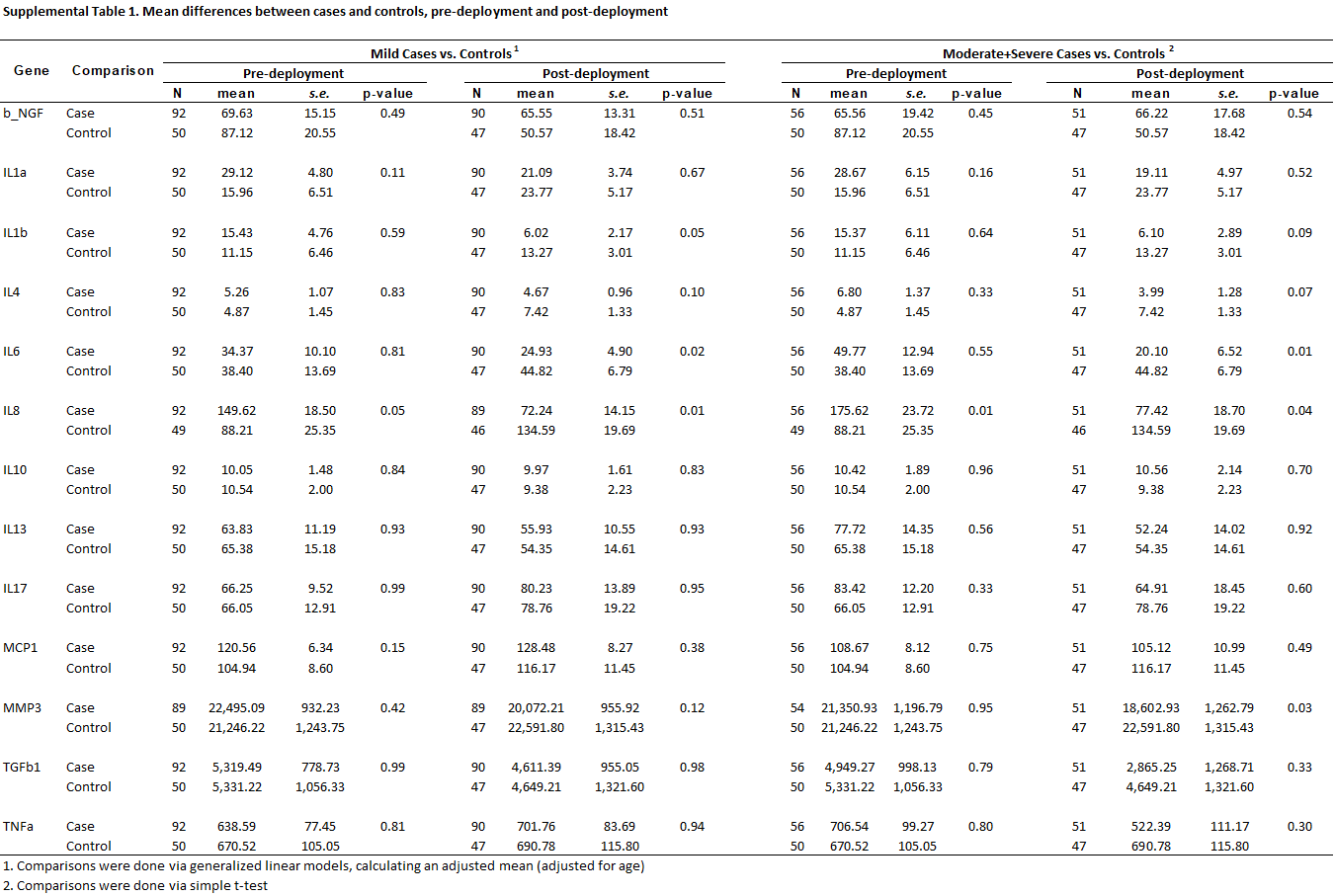


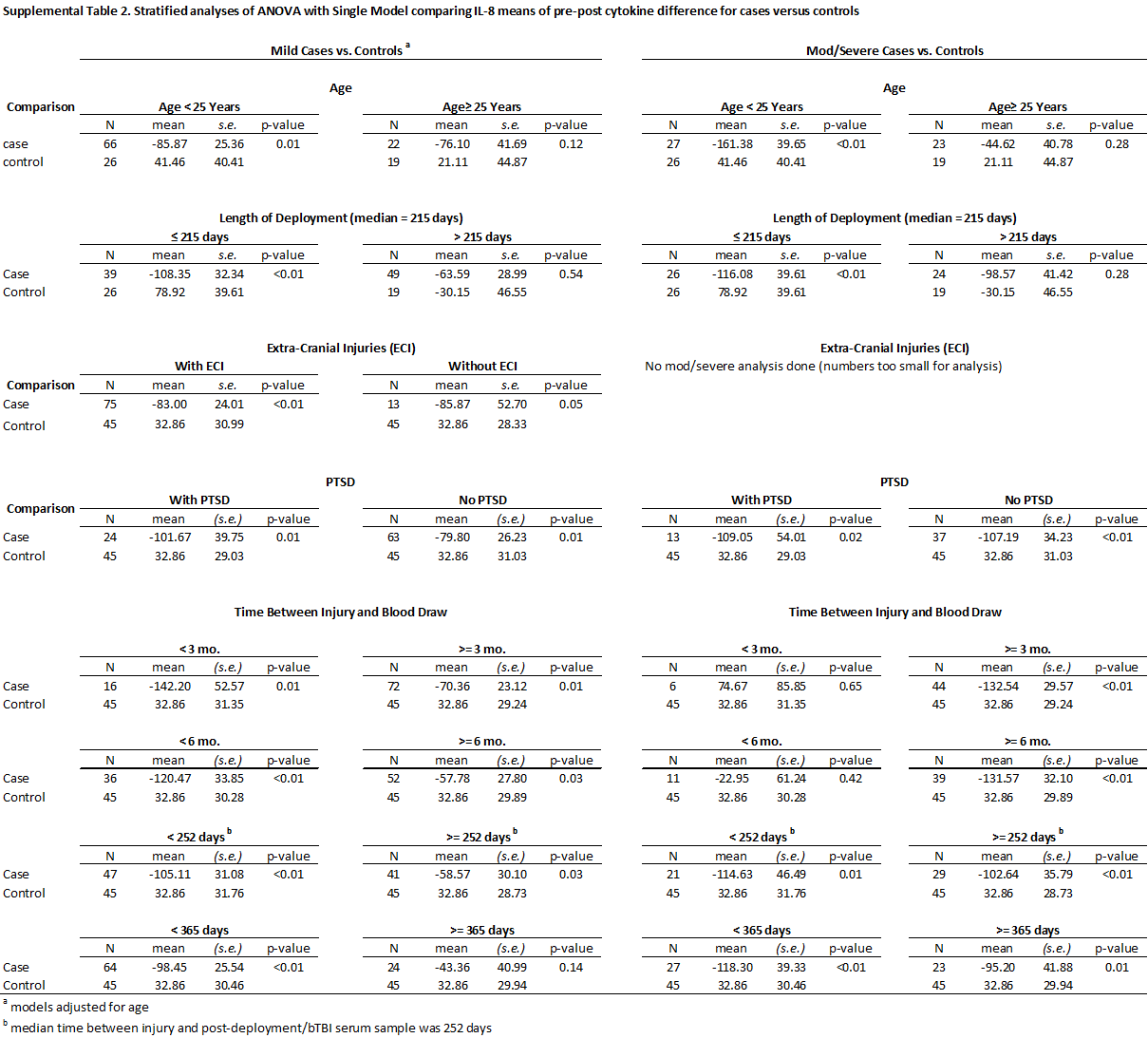

Supplement: Supplementary file 1 — Additional file 1: Table S1. Mean differences between cases controls, pre deployment and post deployment. Table S2. Stratified analyses of ANOVA with single model comparing IL-8 means of pre-post cytokine difference for cases versus controls [file 12974_2019_1624_MOESM1_ESM.docx]
